# Online Social Networks and Smoking Cessation: A Scientific Research Agenda

**DOI:** 10.2196/jmir.1911

**Published:** 2011-12-19

**Authors:** Nathan K Cobb, Amanda L Graham, M. Justin Byron, Raymond S Niaura, David B Abrams, Workshop Participants

**Affiliations:** ^1^The Schroeder Institute for Tobacco Research and Policy StudiesAmerican Legacy FoundationWashington, DCUnited States; ^2^Division of Pulmonary & Critical CareDepartment of MedicineGeorgetown University Medical CenterWashington, DCUnited States; ^3^Department of OncologyGeorgetown University Medical Center / Lombardi Comprehensive Cancer CenterWashington, DCUnited States; ^4^Department of Health, Behavior and SocietyJohns Hopkins Bloomberg School of Public HealthBaltimore, MDUnited States; ^5^see acknowledgements

**Keywords:** Smoking cessation, social support, social networks, addiction, treatment, tobacco

## Abstract

**Background:**

Smoking remains one of the most pressing public health problems in the United States and internationally. The concurrent evolution of the Internet, social network science, and online communities offers a potential target for high-yield interventions capable of shifting population-level smoking rates and substantially improving public health.

**Objective:**

Our objective was to convene leading practitioners in relevant disciplines to develop the core of a strategic research agenda on online social networks and their use for smoking cessation, with implications for other health behaviors.

**Methods:**

We conducted a 100-person, 2-day, multidisciplinary workshop in Washington, DC, USA. Participants worked in small groups to formulate research questions that could move the field forward. Discussions and resulting questions were synthesized by the workshop planning committee.

**Results:**

We considered 34 questions in four categories (*advancing theory*, *understanding fundamental mechanisms, intervention approaches*, and *evaluation*) to be the most pressing.

**Conclusions:**

Online social networks might facilitate smoking cessation in several ways. Identifying new theories, translating these into functional interventions, and evaluating the results will require a concerted transdisciplinary effort. This report presents a series of research questions to assist researchers, developers, and funders in the process of efficiently moving this field forward.

## Introduction

Smoking remains the leading cause of 443,000 preventable deaths and nearly US $200 billion in excess costs in the United States each year [1]. Smoking rates in the United States have stalled near 20%, [2] and large-scale reduction in smoking prevalence remains an urgent public health imperative. Although the evidence-based cessation interventions recommended by the US clinical practice guideline for tobacco dependence treatment [3] have been shown to double quit rates, they are largely underused [4]. Reaching the US public health goals of cutting the smoking rate to no higher than 12% by 2020 [5] will require novel approaches to create new interventions, enhance the effectiveness of existing cessation treatments, and maximize the reach and utilization of both.

The evolution of the Internet and the growth of online social networks may present a solution to the intertwined problems of effectiveness and reach of cessation interventions. Social support [6], social integration [7], and social networks [8] appear to play important roles in smoking behavior and cessation. Yet numerous tobacco treatment studies aimed at creating supportive relationships (eg, peer or buddy training) or harnessing existing social relationships (eg, spouse interventions) have generally yielded disappointing results [9-12]. The limitations of traditional treatment settings in which this work was conducted (eg, low attendance, time constraints, or type and number of available support persons) may partially explain the difficulty in leveraging social support in the cessation process.

Online social networks, by contrast, offer round-the-clock access to vast numbers of participants, potentially superseding these limitations and offering a realistic delivery model for social support. In theory, smokers might benefit not only from active, personal interactions with other network members, but also from various passive sources of social support and influence. Such interactions could alter an individual’s motivation to quit, reinforce the undesirability of smoking, assist in buffering cessation-related stressors and enhancing coping skills, and provide suggestions for eliminating smoking cues [13]. To date, there has been a wealth of behavioral science research on the role of social networks in face-to-face interactions but little published research on online social networks [14-16].

The growth of online social networks and their penetration into popular awareness has been phenomenal, with over 70% of American adults now using some form of social media or online social network [17]. As of early 2011, an estimated 150 million Americans actively use Facebook, the largest of the online social networks [18]. Intentionally created online networks dedicated to smoking cessation are smaller but have been in existence for over a decade. These types of dedicated systems—where smokers and former smokers communicate through various channels in an effort to quit and stay abstinent—are now widely used by hundreds of thousands of smokers over relatively long periods of time [16,19]. Over the years, cessation-focused online networks have evolved from simple systems for the exchange of messages to complex networks complete with multiple modes of communication (eg, chat rooms, forums, or private messaging), self-representation (eg, personal profiles, blogs, or journals), and affiliations (eg, buddy or friends lists, or private groups), through which social norms, social influence, and social support may be conveyed in real time [16,20].

Concurrent with the exponential growth of online social networks has been the rapid evolution of social network science, spurred on as improvements in computer capacity and software have caught up with theory and the burgeoning size of available data sets [21,22]. In studies of real-world networks, social network science has demonstrated that social influence flows through networks and can influence a broad range of behavioral and emotional changes, including smoking and alcohol use [8,16,23], obesity [24,25], happiness [26], and depression [27], as well as loneliness [28] and suicide [29]. Social network analysis allows for an expanded view of an individual’s social universe, taking into account not only their own connections, but also the connections of their friends and contacts and beyond. This ability to look at the social structure in aggregate allows for inferences about how topology (the network structure) both enables and drives behavior change.

Actually cutting smoking prevalence by nearly half by 2020 will require cessation interventions that can reach millions of people in consumer-friendly ways. The convergence of robust evidence for the role of social support in cessation, the growth and proliferation of online networks, and the recent advances in social network analytic techniques present an opportunity for the development and dissemination of high-impact interventions targeting smoking. The notion that online social networks present a powerful and novel approach to cessation is supported by a research in relatively disparate disciplines, including tobacco control, social psychology, and social network science, to name just a few. Leveraging the enormous potential of online social networks to reach and treat smokers will require a transdisciplinary conversation among researchers, developers, and funders that bridges behavioral, network, and computer sciences and other fields [30,31]. We sought to initiate this discussion by convening a multidisciplinary group of experts to identify gaps in knowledge and research questions regarding the potential of online social networks to more rapidly reduce smoking prevalence. Our goal was to construct a strategic research agenda to guide future collaborative work. This paper presents this agenda in the form of 34 pressing research questions and related issues, along with brief discussion.

## Methods

We invited approximately 100 experts and thought leaders (listed under Acknowledgements at the end of this article) across a range of relevant content areas to a 2-day workshop held September 30 to October 1, 2010 in Washington, DC. Participants represented a broad range of disciplines, including economics, engineering, epidemiology, linguistics, mathematics, medicine, nursing, psychology, public health, network science, sociology, software engineering, and product design and commercialization. A small number of participants were invited to give focused overview presentations to help bridge disciplinary borders and to establish a common starting point for discussion. These included presentations on the epidemiology and treatment of tobacco use; basic principles of social support theory and social support interventions in tobacco control; social network science and network-based interventions in tobacco control; the history, evolution, and current state-of-the-science of general and cessation-specific online social networks; and methodological, measurement, and analytic issues regarding social network data collection, analysis, and interpretation. Additionally, representatives from three of the largest for-profit, health-related, online social network interventions were invited to describe their programs and the lessons they had learned in managing online networks.

Following the overview presentations, participants were divided into small multidisciplinary working groups and tasked with developing a list of priority research questions. The guiding framework for workgroup discussions was to address the key question “What do we know and what do we need to learn that will make a difference in improving cessation outcomes?” The framing of the question was deliberately broad to enable participants from diverse disciplines and with varying content expertise to contribute their perspectives.

Participants were instructed to formulate and group research questions into four major categories: (1) *a* *dvancing theory* (developing, refining, or integrating existing theories and models from online and offline social network, social support, smoking cessation, and behavior-change domains), (2) *understanding fundamental mechanisms* (how online social networks produce behavior change at the individual and network level), (3) *intervention aproaches valuation* (methods and metrics for appropriate program, process, and outcome evaluations). To encourage brainstorming, groups were instructed to imagine they had access to the intellectual, financial, and technical resources represented by any combination of the attendees or speakers at the workshop. Each working group presented their list of research questions back to the full group for further discussion. The groups worked independently on each major theme area, and then their recommendations were synthesized and refined with feedback from the entire group. After the workshop, the planning committee met to review the findings and to summarize the general areas of research topics and priorities for dissemination.

## Results

Participants generated a large number of research questions at varying levels of granularity. Common and overlapping ideas were integrated and a subset of questions was selected for further discussion and elaboration by the report’s authors. For each key topic area, we present a summary of discussions and provide examples of the most pressing research questions or issues raised.

Several overarching themes emerged from the discussions. First, participants noted that traditional models of offline (eg, face-to-face) intervention and evaluation are often reflexively applied to online observations or interventions. While there are ways in which offline and online behaviors overlap and can reciprocally inform models, mechanisms, implementation, and evaluation, there are also important differences that require critical thinking about online networks. There is a need to challenge and test the assumptions inherent in traditional models when developing, implementing, and evaluating online interventions.

A second theme related to the mechanisms of behavior change. Numerous theory-based processes of behavior change have been described within social networks, including diffusion of information, viral spread of interventions, social support, social norms, and modeling. It is unknown whether these or other unidentified processes are important in online social networks for cessation, and if any of these may be iatrogenic (ie, promoting continued smoking rather than cessation).

A third theme centered on the appropriate use of theoretical models, empiricism, and statistical or simulation modeling techniques. Future advances in online social network interventions will likely depend on a transdisciplinary approach to develop appropriate theoretical models, test them in vivo and in silico (software modeling), rapidly iterate to determine interventions with the highest probability of effect, and perform intervention trials with appropriate research designs and end points. Such advances will require improvements in existing capacity to collect complex and large-scale longitudinal data on behaviors and interactions within online networks.

Finally, we note a common assumption during the workshop that social network interventions will increasingly take advantage of mobile delivery mechanisms—whether smart phone apps, text messages, or other formats. While few questions address this shift explicitly, we have attempted to write this summary to be agnostic toward delivery platform. Both questions and recommendations are intended to be broadly applicable, regardless of location or modality.

### Advancing Theory of How Social Networks Influence Smoking-Cessation Behavior

Social network and social support models in smoking cessation [6] derive primarily from social learning theory [32] and from the study of interventions to change existing social support interactions or develop new supports (eg, from a counselor and other members of a group participating in face-to-face smoking-cessation treatments) [10,33]. Observational studies have shown that social support is associated with smoking initiation and cessation, and that smokers associate with other smokers in proximal social networks. Intervention studies, though, have yielded mixed and largely disappointing results in attempting to manipulate or harness support [6,10,33]. These findings highlight the importance of the need for a theoretical framework that permits simultaneous understanding of the observed association between social phenomena and smoking, and manipulation of the social environment to effect change. There is a need to evolve our theories and models to refine their explanatory power and their applied utility to facilitate behavior changes, such as smoking cessation [34].

The application of network theory to social networks has largely occurred in studies of real-world (ie, offline) networks [8,29,35-37] using retrospective self-report measures and cross-sectional data. Recent research on social networks has rapidly evolved using online data [16,38-42], new computational methods, and mathematical and simulation modeling [37,43,44]. Data from electronic communications networks (eg, online social networks, email systems, and telephone networks) can be collected in real time and can record communications and interactions at multiple levels and with repeated observations of intraindividual, interindividual, and contextual influences. Such methods can inform interventions and measures of process and outcome, but the proliferation of data and results calls for new or more refined models or theoretical frameworks to facilitate interpretation and application.

Theories that try to explain and change behavior in small real-world settings may not translate easily into the online world, where interactions occur on a larger scale and in a different medium. A transdisciplinary synthesis [30,45] is likely required to integrate our understanding of the nature and form of social networks, as instantiated or reflected in the online world, and their functions that serve to initiate and maintain changes in individual behaviors. Structuralist social network theories, which address how patterns of social relationships are associated with substantive topics such as health behaviors [37,45], come closest to fusing form and function, and serve as a useful point of departure for understanding how social networks and individual tobacco use behaviors intersect.

A century ago, Simmel [35] called for more than knowing how to measure characteristics of networks, such as the density of their interconnections, and recommended developing a set of assumptions about how best to describe and explain the social phenomenon of interest in its proper context. This challenge remains today as we seek to integrate social and individual theories of behavior change. Indeed, the structure of a network may induce, maintain, or strengthen a behavior not just by transmission of information, but via forces of exclusion, adaptation, or the binding together of members [46]. Looking at network causes of phenomena of interest requires asking what kinds of social networks lead to particular outcomes.

### Specific Questions

1. How well do theoretical models of social influence translate between offline and online contexts?

2. How does online social network data map onto real-world networks? Does research based on retrospective self-report with sparse observations in the real-world match with dynamic, observed behavioral data collected online?

3. How can behaviorally important ties be identified in online social networks that may be composed of large numbers of apparently weak ties?

### Understanding Fundamental Mechanisms

Online social networks exist across a broad range of health conditions and behavioral risk factors including tobacco use (eg, becomeanex.org, quitnet.com, and stopsmokingcenter.com), diet and fitness (sparkpeople.com), diabetes (tudiabetes.com), chronic diseases (patientslikeme.com), and others [16,47-49]. Research is in its infancy regarding the mechanisms through which these online social networks might or might not effect behavior change. Social support models suggest that behavior change is mediated in part through information exchange, instrumental or emotional support, stress buffering, or improved self-efficacy [6]. However, other mechanisms may be as or more important in online networks, such as exposure to new or different norms or behaviors modeled by other network members [16]. To date, the design and implementation of online networks has been largely based on offline cessation approaches, usually comprising only small groups of smokers actively trying to quit. The evolution of more effective cessation interventions will require an in-depth, sophisticated understanding of the unique aspects of online social networks and the specific mechanisms through which they effect behavior change, as well as the careful selection of evaluation strategies matched to this intervention context.

#### Homophily, Heterophily, and Network Topology

Homophily refers to the tendency of people to associate with similar others (“birds of a feather”), while heterophily refers to the tendency to collect in diverse groups. That homophily tends to be a driving factor in the formation of social networks [50] is an important consideration in offline networks: the tendency of smokers to associate with other smokers may decrease the impact of normative exposure to nonsmokers or former smokers within a network. In contrast, online social networks may be heterophilous, comprising individuals across the cessation continuum including individuals who have been abstinent for years [16] or current smokers who are curious but not yet motivated to quit. Research in offline networks suggests that topological factors (the pattern of ties between individuals within the network), such as clustering of smokers, affects cessation over time [8]. Other work in online networks indicates that dense connections at the individual level reinforce social signaling and increase the chance of behavior change [39]. As most existing online networks remain uncharacterized, little is known about their structure or the optimal topology to effect behavior change.

##### Specific Questions

1. What is the role of homophily in the formation of online networks?

2. What is the role of heterophily in the provision of social support throughout the cessation process, and how does it influence cessation outcomes?

3. Can ties within online social networks be fostered or manipulated to “rewire” networks, modify topology, and drive behavior change?

4. What impact does network topology have on behavior change? For example, does having a dense local network increase the probability of making a quit attempt, cessation, and maintenance of abstinence?

#### Social Diffusion

Information and behavior diffusion through offline social networks are well-studied phenomena, encompassing myriad behaviors from seed choice by farmers to the spread of smoke-free policies from city to city [51]. In contrast, inducing or manipulating diffusion through both online [40,41] and offline networks [51,52] has proved challenging in practice; deliberately causing spread of information or a behavior is easier to conceptualize than to implement. In commerce and industry, the term viral marketing refers to this deliberate seeding and resulting diffusion of a message through a targeted network, such as the promotion of a new product [53]. While viral marketing is a common practice, there is little academic literature on its use for health topics or for online approaches for health behavior change. Nonetheless, deliberate seeding and diffusion may allow for the dissemination through created networks of specific information (eg, information about a new cessation medication), interventions (eg, a quit smoking app through Facebook), smoking/cessation norms, and other health behaviors.

##### Specific Questions

1. How does information spread through an online social network? Are there identifiable patterns of information spread that can be leveraged in intervention research?

2. Can key participants in a network be identified and targeted to foster information diffusion or make it more efficient?

3. What are the drivers of the viral spread of an application, concept, or innovation through online networks?

4. How does network topology affect diffusion? Can social network measures and concepts such as centrality or clustering be used to predict or alter diffusion?

#### Social Norms and Modeling Behavior

Despite the fact that members do not know each other at the outset, created online networks can develop their own language and norms [54]. Existing members may convey expectations for certain behaviors or participation in the network that guide and support new or struggling members [55]. These expectations and norms may differ from those in the participant’s offline network. For example, the public health community has worked hard to normalize the use of nicotine replacement as a cessation aid; however, most smokers do not use pharmacotherapy when quitting [4] and many have concerns about the safety of any form of nicotine [56]. Online social networks may present norms supportive of medication use, and existing users may model successful medication use behavior. Other norms such as recycling after failed quit attempts, enlisting external social support, or the use of telephone counseling are other examples of potential norms (positive or negative) within social networks.

##### Specific Questions

1. How are social norms established and communicated in an online social network?

2. What is the effect of online social norms when they differ from a user’s offline environment?

3. Does anonymity in online networks enhance or diminish the effect of modeling behavior and communication of norms?

4. Are norms and modeling effective mechanisms to influence “lurkers” (ie, members of a network that read other members’ posts/comments but rarely communicate with other members)?

#### Network Formation, Social Integration, Retention, and Longitudinal Stability

There are numerous online communities and created social networks dedicated to health-related behavior change—some of them in existence for over a decade with thousands of members—yet it remains unclear what factors led to their growth or stability. Previous research has shown that small numbers of individuals may be responsible for approaching and “integrating” new members as they join an online network for cessation [16]. Most research has reported results from successful, stable networks [14,16,57], while projects that fail to form networks are rarely reported [58]. As a result, the factors that drive member integration and retention and network stability remain unclear. Adequate understanding of these factors is required to build new interventions and to maintain existing versions or enhance their effectiveness.

##### Specific Questions

1. What predicts engagement in an online social network? What demographic, smoking, psychosocial, or other characteristics are predictive of participation and integration?

2. What is the role of timing of interactions in online social network in influencing integration and participation? What forms of outreach and communications (eg, private messages, instant messaging, public forums, or blogs) drive tie formation?

3. What is the role of long-term users in network structure and network stability over time?

### Intervention Design and Approaches

The incredible growth of online social networks offers the opportunity for novel intervention designs. Created networks such as online communities dedicated to smoking cessation are a common component of modern health behavior-change systems and often center on the “build it and they will come” premise of intervention delivery. These networks generally comprise motivated individuals ready to make or maintain changes to one or more health risk behaviors. Such systems benefit from a specific focus, on the part of both the user and intervention designers. However, they generally do not yet take full advantage of the potential to proactively reach larger populations. Individuals must generally seek out and enroll in the closed system, and ultimately many registrants fail to return to the site [59], much less engage with the social aspects as designed (they become, at best, lurkers or, at worst, completely unengaged). In contrast, general-purpose networks such as Facebook offer unique opportunities and challenges, related primarily to their enormous size, including the potential for autonomous propagation (viral spread) of interventions. Certainly, intervention design decisions should be informed by relevant and sophisticated theories that specify the active ingredients and mechanisms of action, but the surfeit of potential participants in these extremely large networks ultimately allows for data-driven methods to drive the ongoing design and refinement of interventions.

#### Target Populations

Smoking-cessation interventions most frequently target individuals ready to make a quit attempt. Yet many people who join online cessation systems have already quit or are not ready to make a quit attempt [60-63]. Traditional social support models will need to be modified to assist these individuals and to maximize their utility in supporting others. Significant public and private resources are used each year to denormalize smoking and encourage cessation using traditional media [64,65]. As public health organizations increasingly use advertising and outreach efforts to drive utilization of online resources, it will become imperative to identify the types of smokers that may benefit from social network-based approaches to cessation. A one-size-fits-all approach is unlikely to be efficient or effective, and it is unclear how much customization or individual tailoring is needed to make an incremental addition to outcomes [66,67].

##### Specific Questions

1. Do smokers who are not motivated for behavior change benefit from social network interventions? What influence do social support and normative exposures have on smokers who may not be thinking of quitting?

2. Can online social networks assist smokers who have already quit to maintain abstinence? Can recruiting abstinent smokers into a network strengthen the network’s capacity for social support?

3. Are demographic or psychosocial characteristics important predictors of online social network utilization? What is the impact of age, gender, race/ethnicity, or other identifying characteristics with regard to network phenomena such as integration or tie formation?

4. How can network-based interventions capitalize on secular trends and historical events, such as a change in the federal excise tax rate, new year’s resolutions, The Great American Smokeout, or major smoking-related media stories such as the death of Peter Jennings from lung cancer? Do smokers recruited during the “surges” associated with these events differ from those who join an online social network at other times or for other reasons?

#### Systems Integration

The oldest examples of online social networks for cessation are relatively siloed intervention approaches, focused largely on engaging users with other participants on a cessation-specific website and in an anonymous fashion. More recent interventions integrate online social networks into other treatment-delivery approaches, such as telephone quitlines [13,68,69]. The rapid expansion of large-scale networks such as Facebook where users are personally identifiable offers the opportunity to disseminate cessation interventions through existing networks, but without the aspect of anonymous participation. It is unknown the degree to which the advantages of leveraging an existing network where participants are identified are offset by the potential benefits of a network where members are anonymous. Integration of online social networks with other treatment modalities (eg, text messaging, health care-delivery settings, or electronic medical record systems) offers the opportunity to enhance treatment effectiveness, augment social support mechanics, and increase the reach of traditional services. At the same time, such integration introduces multiple new complexities.

##### Specific Questions

1. What is the best mechanism for online social networks to interface with other elements of health care or tobacco treatment (eg, telephone quitlines, over-the-counter and prescription pharmacotherapy, physician advice, electronic medical record, mass media campaigns, or policies)?

2. How does involving a smoker’s offline network (eg, friends, family, medical practitioners, worksite wellness, or occupational health programs) augment or diminish the effect of an online social network on cessation?

#### Development Methods

There is a chasm between the rapid-cycle, diffusion-focused development methods used by entrepreneurs and industry to launch online programs and the traditional, efficacy-based development methods of behavioral and social scientists. For example, Facebook has grown literally from a dorm room project to over 150 million Americans a month in approximately 6 years. Ironically, this is typically the same amount of time between submission of a federal grant application and the publication of its main outcome paper. Shortening this timeline is critical if we are to develop effective interventions that can be deployed on a large scale to benefit public health in a timely fashion. Engineering principles of iterative development and early evaluation have been adapted in the behavioral sciences (eg, multiphase optimization strategy, or “MOST”, [70]) and provide one approach to achieve this goal. Online interventions are particularly suited to these methods; large, available target populations enable intervention variations to be tested against each other with statistical significance in rapid sequence or in factorial models, in theory improving effectiveness and tightening research and development timelines [67,70].

##### Specific Questions

1. Can engineering models, such as MOST, speed development time and/or increase efficacy of network-based interventions?

2. What process and outcome metrics are most appropriate during intervention development and refinement? Participant engagement? Retention? Network integration? Quit attempts? Early abstinence?

### Evaluation

Several high-quality randomized controlled trials of Internet cessation programs have been conducted [13,57,62,67,71-76]. Yet, to date, there have been no published reports of tobacco intervention trials that link social network structure or dynamics to either social support metrics or more distally to cessation outcomes. Not all of these trials have included social network components, but among the ones that did, there are several reasons for this gap in the literature: the difficulty of constructing appropriate assessment and intervention protocols, the difficulty in maintaining participants in social interventions, and the challenge in disentangling social processes from other features of many Web-based interventions (eg, tailored materials, expert systems, or access to counseling staff). There is a critical need for the identification of appropriate research designs, data collection methods, and evaluation strategies to determine the impact of social processes within online interventions that may drive cessation and abstinence.

#### Research Design

The use of randomized control trials in research to evaluate online social network-based interventions presents a number of challenges. Among these are selecting a feasible, ethical, and rigorous control condition [77,78]; avoiding contact between participants randomly assigned to different conditions; and managing the attrition observed across virtually all online interventions [59]. Alternative evaluation designs used in eHealth research, such as practical clinical trials, pragmatic randomized controlled trials, and nonexperimental and quasi-experimental designs [77,79-81], may be appropriate as well for social network interventions. Given the size of data sets that are generated from online social network interventions, automated systems for the categorization and extraction of data (such as natural language processing and sentiment analysis, data mining, and pattern recognition) may also play important roles in exploratory analyses. The use of varied methods and data sets will make consistent and standardized reporting of results increasingly important as the field advances.

##### Specific Questions

1. Given that alternative Internet interventions are a mouse-click away, what are the important considerations in selecting a rigorous and appropriate control group and evaluating contamination (ie, exposure to the intervention arm among control participants)?

2. Other than randomized control trials, what rigorous research designs can be aptly used to optimize online social network interventions? Are there specific research designs that are best used at specific phases of the development–dissemination–implementation continuum?

#### Data Collection and Analysis

Online interventions and social networks in particular are part of the “big data” problem [22], an emerging issue where the quantity of behavioral and other process data exceeds the capacity for traditional analysis. Academic computational social science—the collection and analysis of these data—lags behind other fields such as physics and biology, as well as the corporate capacity of Google and Facebook in managing big data [21]. The two primary challenges inherent in big data are adequately defining and capturing the appropriate data, and conducting effective and efficient analyses. Data collection methods such as ecological momentary assessment, mobile tracking data, content and sentiment analysis, and observation of online interactions can provide granular information about behavior with minimal impact on the user or their friends and contacts. These methods can generate much richer—and also more complicated—representations of social networks that contain information about the weight of ties, their valence (positive or negative), and the presence of hidden or latent ties [82].

##### Specific Questions

1. How can novel data collection methods such as ecological momentary assessment, passive tracking data from websites, or data from mobile devices be used to gather network-level data without affecting individual behavior or the network itself?

2. What new techniques and analytic methods will be required for analysis of “big data” and increasingly complicated network representations?

#### Expanded Outcomes and End Points

Traditionally, research has evaluated the impact of an intervention only on the individuals enrolled in a study. Bolstered by evidence from both offline [8,23-29,51,52] and online [39-42,53] studies, network theory suggests that behavior change may diffuse through a network. Successful intervention with an individual smoker may have positive externalities (a term for collateral effects, drawn from the economic literature) that ripple through the network [83] causing other smokers to quit or to cut back on their smoking, or resulting in changes in attitudes or other beliefs [8]. For example, a quit attempt by an individual enrolled in a program might prompt a close friend to also attempt cessation. Success of the friend would not normally be part of a traditional analysis, but becomes critical from a network standpoint, particularly since interventions may be specifically designed to elicit this effect. Given that evaluating changes in behavior among individuals outside the purview of a research study may be difficult or impossible, alternative end points, outcomes, and evaluation strategies become imperative [83].

##### Specific Questions

1. What end points or surrogate outcomes will permit the evaluation of externalities in online network interventions?

2. What are the ethical implications of observing or even inducing behavior change in individuals that have not consented to participate in a research study?

#### Modeling to Inform Design and Evaluation

The use of mathematical predictive models in public health, and tobacco control in particular, has recent support [84-86]. Their use to design, refine, or evaluate behavioral interventions for cessation is less defined, but the opportunities are compelling. Previously, models have been employed to examine how best to optimize the multiple modes of delivery of smoking-cessation interventions, as well as to capitalize on context, such as multilevel influences of restrictive polices, mass media, and increased sales taxes [85,87-89]. In silico techniques such as agent-based modeling, where powerful computers simulate autonomous users interacting within a network over time, can be used to predict responses to intervention design changes [90]. Under certain circumstances they can also be used to disentangle behavioral outcomes from network processes and potentially contribute to evaluation [34]. Such techniques not only may play a valuable role in accelerating intervention development and evaluation, but also may help to determine the potential impact of interventions prior to time consuming and costly promotion and implementation–dissemination efforts.

##### Specific Questions

1. How can mathematical and computer-driven simulations of various kinds (eg, dynamic systems models or agent-based models) contribute to intervention development, refinement, or evaluation?

2. How can existing systems models inform work with online social networks? How might existing systems models be affected or informed by large-scale social networks (such as Facebook)?

## Discussion

An increasingly interconnected online social Web provides incredible opportunities to shift behavior, affect health, and meet public health challenges. Despite promising starts in individual fields, it will take further rigorous and transdisciplinary research and development to meet the potential described in this report. Tackling the questions posed here, structuring research protocols, and developing appropriate analytic techniques will require true collaboration across multiple fields and divergent disciplines [30]. Success may lead to interventions with the capacity to reach large populations, augment existing treatment modalities, and effect behavioral change in novel ways.

While we have focused on tobacco use and smoking cessation, the same questions and approaches may apply to virtually any behavior change of interest. Interventions need to be informed by and should inform theory, model testing, and protocols for refinement. As we gain experience working in transdisciplinary teams and refine our models, we will have a clearer picture of the new measures needed for empirical data collection and testing of models to identify the mechanisms, pathways, and key processes that influence intermediate and final behavior-change outcomes of interest. Such iterative approaches will also lead to ways to validate self-report measures and integrate or triangulate the tracking of online activities with observational data and social network and support activities that are conducted offline.

Given the rapid evolution of the field of online communications and smoking-cessation interventions, and the numerous disciplines involved, we will need more agreement and standardization on metrics. For example, assessing norms and answering questions about their impact on behavior will require the development and validation of new instruments to determine active norms in an online social network and their importance. This work will be a necessary precursor to any efforts to modify existing norms or introduce new norms into existing or evolving networks. Ultimately the refinement of theories, models, and interventions would benefit from the development of standardized measures not only for norms, but for virtually all metrics mentioned in this report. Such measures would ideally have good reliability and validity across different projects, organizations, and even disciplines. Establishing a set of core measures that should be used across studies of online social networks will help test and improve both internal and external validity and will enhance theory testing by ensuring robustness, generalizability, replicability, consistency, and convergent validity across studies.

There are several limitations to this report. The recommendations presented are dependent on the individuals present at the conference and the structure provided by the organizers. Different participants or a different structure undoubtedly would have produced different questions and topics. The research priorities and recommendations presented here are but one set of views that we hope will serve to stimulate additional dialogue and research efforts. Addressing the questions posed in this report will present significant, but not insurmountable, challenges around personal privacy and the ethical treatment of research participants and their social contacts. Behavioral and biomedical researchers have traditionally thought about the impact on individuals, but social network interventions will challenge us to draw on the experience of public health professionals, social marketers, and sociologists as we increasingly target networks.

Networks and technology evolve on their own timeline, independent of the needs, funding, or aims of researchers. The study of rapidly evolving networks will require investigators and funders to tighten their timelines through the entire process (from idea, to funding, to execution, to publication). The traditional models of funding research via federal grants such as those in place at the National Science Foundation or National Institutes of Health in the United States are notoriously slow compared with industry and entrepreneurial interests. Network science and online interventions are changing rapidly and the traditional funding models must adapt as well. In 2009, Lazer and colleagues voiced concerns that research on large-scale networks “could become the exclusive domain of private companies and government agencies” [21], an outcome they noted would not be in the public interest. Developing and maintaining a strong academic research program is imperative and will require adjustments by funders, researchers, and publishers of scientific research.

Research efforts designed to address the topics and questions in this report may help identify mechanisms to significantly decrease the burden of tobacco related disease in the United States and elsewhere. The core ideas and themes developed here for smoking cessation may also apply—recognizing differences in context—to a variety of behaviors (eg, obesity, substance abuse, or adherence to medical recommendations) that could directly or indirectly improve the well-being and quality of life of our society. It is important to recognize that the powerful forces and rapid transmission of information across networks may also be used inappropriately or destructively (both intentionally and unintentionally) as well as for doing good. Ultimately, we hope that the kinds of research efforts encouraged in this paper will give rise to a new generation of interventions to help people quit smoking and stay quit, delivered and spread through a variety of social networks—networks that we recognize today, and networks that will develop tomorrow.

## Multimedia Appendix 1

Conference introduction, Dr. David Abrams.

## Multimedia Appendix 2

Conference introduction - Dr. Saul Shiffman.

## Multimedia Appendix 3

Theme  1: Social  support,  health  behavior  and  smoking  cessation - Dr. Robin Mermelstein.

## Multimedia Appendix 4

Theme  1: Social  support,  health  behavior  and  smoking  cessation - Dr. Thomas Valente.

## Multimedia Appendix 5

Conference keynote - Dr. Nicholas Christakis.

## Multimedia Appendix 6

Theme  2:  Online  social  networks - Dr. Nathan Cobb.

## Multimedia Appendix 7

Theme  2:  Online  social  networks - Dr. Noshir Contractor.

## Multimedia Appendix 8

Theme  2:  Online  social  networks - Dr. Nathan Eagle.

## Multimedia Appendix 9

Theme  3:  Intervention  approaches - Mr. Dave Heilmann.

## Multimedia Appendix 10

Theme  3:  Intervention  approaches - Mr. Trevor va Mierlo.

## Multimedia Appendix 11

Theme  3:  Intervention  approaches - Mr. Chris Cartter.

## Multimedia Appendix 12

Theme  4:  Methods, design  and  analysis - Dr. Linda Collins.

## Multimedia Appendix 13

Theme  4:  Methods, design  and  analysis - Dr. Tom Snijders.
