# Metadata Correction: Online Social Networks and Smoking Cessation: A Scientific Research Agenda

**DOI:** 10.2196/jmir.2049

**Published:** 2012-01-11

**Authors:** Nathan K Cobb, Amanda L Graham, M. Justin Byron, Raymond S Niaura, David B Abrams, Workshop Participants

**Affiliations:** ^1^The Schroeder Institute for Tobacco Research and Policy StudiesAmerican Legacy FoundationWashington, DCUnited States; ^2^Division of Pulmonary & Critical CareDepartment of MedicineGeorgetown University Medical CenterWashington, DCUnited States; ^3^Department of OncologyGeorgetown University Medical Center / Lombardi Comprehensive Cancer CenterWashington, DCUnited States; ^4^Department of Health, Behavior and SocietyJohns Hopkins Bloomberg School of Public HealthBaltimore, MDUnited States; ^5^see acknowledgements of original article

## Authorship Correction

The authors of "Online Social Networks and Smoking Cessation: A Scientific Research Agenda" (J Med Internet Res 2011;13[4]:e119) inadvertently omitted Raymond S. Niaura from the list of authors during the submission process. The author Niaura should have been added after M. Justin Byron in the originally published manuscript. This error has been corrected in the online version of the paper on the JMIR website on January 11, 2012, together with publishing this correction notice. This was done before submission to Pubmed Central and other full-text repositories, but after submission to PubMed/Medline. Correction of the Pubmed/Medline record will be requested and will be done by the National Library of Medicine (NLM). The Crossref/DOI record has been corrected by the publisher.

